# Perceived Shared Decision Making during Maintenance Hemodialysis: Associations with Health Literacy, Social Deprivation, and Care Setting

**DOI:** 10.34067/KID.0000001214

**Published:** 2026-05-04

**Authors:** Abdallah Guerraoui, Najoi Elkalai, Fitsum Guebre-Egziabher, Agnès Caillette-Beaudoin, Julie Haesebaert

**Affiliations:** 1 Calydial, Vienne. Hospital Center Lucien Hussel, Montee Dr Maurice Chapuis, Vienne, France; 2 Research on Healthcare Performance RESHAPE, INSERM U1290, Université Claude Bernard Lyon 1, Lyon, France; 3 Department of Nephrology, Hôpital Edouard Herriot, Hospices Civils de Lyon, INSERM U1060 CarMeN Laboratory, Université Claude Bernard Lyon 1, Lyon, France; 4 Hospices Civils de Lyon, Pôle Santé Publique, Service Recherche et Epidémiologie Cliniques, Lyon, France

**Keywords:** health equity, diversity, and inclusion, hemodialysis, patient self-assessment, patient-centered care, home hemodialysis

## Abstract

**Key Points:**

- Perceived shared decision making during routine hemodialysis care was low and heterogeneous across patients.
- Higher communicative and critical health literacy and lower deprivation were associated with higher perceived shared decision-making.
- Dialysis autonomy and unit type were associated with perceived shared decision making, suggesting organizational targets for patient-centered care.

**Background:**

Shared decision-making (SDM) is a core component of patient-centered care. In CKD, SDM research has largely focused on dialysis modality choice, with limited attention to routine clinical decisions during maintenance hemodialysis. We assessed patients' perceived SDM during routine hemodialysis care and examined individual and contextual factors associated with perceived SDM.

**Methods:**

A multicenter, cross-sectional, observational study was conducted among adult patients receiving maintenance hemodialysis at the Centre Associatif Lyonnais De Dialyse dialysis network in the Rhône-Alpes region, France. Data were collected during routine dialysis sessions over a 2-week period from June 24 to July 6, 2024. Independent variables included individual characteristics (age, sex, education, health literacy [HL]), socioeconomic deprivation (European Deprivation Index), dialysis autonomy, dialysis unit type, and decision characteristics. Perceived SDM was assessed using the nine-item SDM Questionnaire (SDM-Q-9). Univariable analyses and multivariable linear regression were performed to identify factors associated with SDM-Q-9 scores.

**Results:**

Among 104 participants (75% male; 88% older than 50 years; 64% retired), the mean SDM-Q-9 score (0–100 scale) was 24.3±26.1 (median, 13.3), indicating low perceived SDM. Most reported decisions concerned dialysis management (57%), followed by medication-related decisions (26%) and comorbidity management (17%). In multivariable linear regression analysis, higher communicative (*β*=2.48; 95% confidence interval [CI], 0.89 to 4.07; *P* = 0.003) and critical HL (*β*=3.01; 95% CI, 1.12 to 4.90; *P* = 0.002) were associated with higher SDM-Q-9 scores, whereas greater socioeconomic deprivation was associated with lower scores (*β*=−1.12; 95% CI, −2.05 to−0.19; *P* = 0.02). Dialysis unit type, dialysis autonomy, and employment status were also associated with SDM-Q-9 scores.

**Conclusions:**

Perceived SDM during routine hemodialysis care was generally low and heterogeneous. HL, socioeconomic deprivation, dialysis autonomy, and care setting were associated with perceived SDM. Dialysis vintage was not recorded and not included in the analyses. Interventions addressing HL-sensitive communication and organizational factors may improve patient-centered decision making in hemodialysis care.

## Visual Abstract

**Figure:**
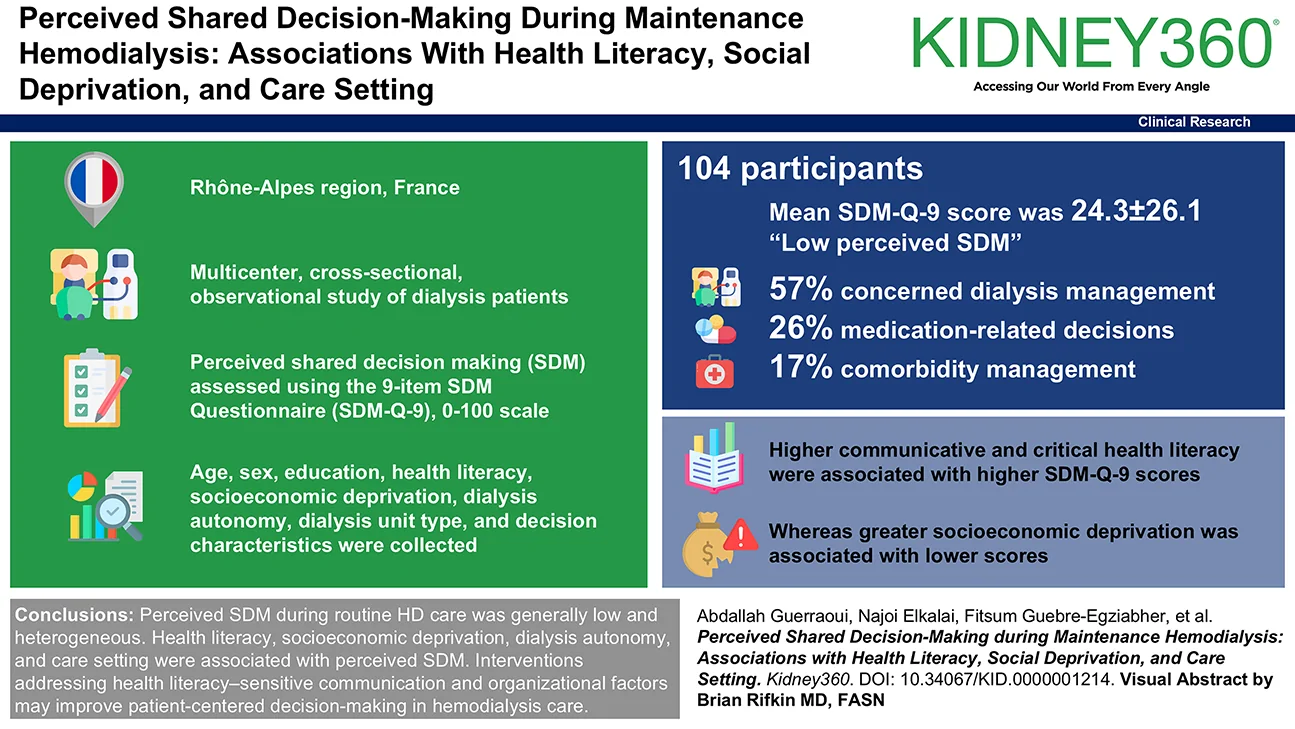

## Introduction

Shared decision-making (SDM) is a key component of patient-centered care (PCC) and is increasingly promoted as a standard of high-quality health care.^1,2^ SDM refers to a collaborative process in which physicians and patients work together to make health care decisions, combining the best available evidence with patients' values, preferences, and lived experiences.^1,3^ This approach is particularly relevant in chronic diseases, where treatment decisions are complex, recurrent, and closely linked to patients' daily lives.^2^

In nephrology, SDM has primarily been studied in the context of dialysis modality choice and advance care planning.^4–7^ However, patients receiving maintenance hemodialysis face frequent routine decisions, including adjustments to dialysis prescriptions, medication changes, and the management of comorbid conditions.^8,9^ Such routine decisions can have a substantial effect on symptoms, quality of life, and treatment burden, yet little is known about how patients perceive their involvement in these everyday clinical decisions.^10^ SDM is also relevant in routine hemodialysis care, where repeated decisions (such as dry weight adjustments, antihypertensive or phosphate binder prescriptions, and symptom management) influence clinical outcomes, treatment adherence, and polypharmacy burden.^8,9^ Incorporating patients' preferences and experiential knowledge into these day-to-day decisions may therefore represent an important mechanism through which SDM can contribute to improved clinical and patient-reported outcomes. Previous studies suggest that patients' engagement in SDM varies widely and is associated with individual and contextual factors such as age, education, and clinical setting.^11,12^ Moreover, systematic reviews have identified persistent barriers to the implementation of SDM in routine care, including time constraints, perceived patient vulnerability, and organizational limitations.^13^ Evidence on the effectiveness of interventions to increase SDM in everyday clinical practice remains limited, highlighting the need for observational studies that describe real-world SDM experiences from the patient perspective.^13^

Health literacy (HL), defined as an individual's ability to obtain, understand, and use health information to make informed decisions, is likely to play an important role in SDM, particularly in hemodialysis populations.^14,15^ Managing ESKD requires patients to understand complex medical information, adhere to demanding treatment schedules, and participate in ongoing decision making.^16,17^ In populations receiving maintenance hemodialysis, where treatment regimens are complex and decisions are recurrent, limited HL may further constrain meaningful participation in SDM.^18,19^ Limited HL among patients on hemodialysis has been associated with poorer clinical outcomes, including higher hospitalization rates, missed dialysis sessions, and increased mortality.^20–23^ Understanding how HL relates to patients' perceived involvement in decision making may therefore help identify opportunities to improve communication and care delivery, particularly for patients treated in underserved areas, who may face additional structural and contextual barriers to patient engagement. The objective of this study was to assess patients perceived SDM during routine hemodialysis care and to identify associated individual and contextual factors, including HL, socioeconomic deprivation, and care setting.

## Methods

### Study Design and Settings

A multicenter, cross-sectional questionnaire-based observational study was conducted among adult patients receiving maintenance hemodialysis. Data were collected during routine dialysis sessions over a 2-week period from June 24 to July 6, 2024, across all hemodialysis units of the nonprofit Centre Associatif Lyonnais De Dialyse dialysis network in the Rhône-Alpes region, France. No *a priori* sample size calculation was performed. All eligible patients treated in the participating dialysis units during the 2-week inclusion period were consecutively invited to participate; the study should therefore be interpreted as exploratory and hypothesis-generating.

Dialysis settings were categorized into three predefined unit types according to patient autonomy: (*1*) center-based hemodialysis units, providing fully assisted in-center treatment; (*2*) medicalized dialysis units (UDM), offering center-based hemodialysis units with a higher level of nursing supervision and limited on-site physician presence (semiautonomous); and (*3*) self-dialysis units, in which patients performed dialysis with varying degrees of assistance from nursing staff (autonomous). The Centre Associatif Lyonnais De Dialyse network includes two center-based hemodialysis units located in Vénissieux and Vienne; three UDMs located in Vénissieux, Vienne, and Pierre-Bénite; and two self-dialysis units at Vienne and Pierre-Bénite. The dialysis units serve populations living in territories with varying levels of social deprivation, measured using the European Deprivation Index (EDI). EDI values were assigned at the dialysis-unit level and reflect the socioeconomic characteristics of the geographic areas served by each unit (Pierre-Bénite≈6, Vienne≈6, and Vénissieux≈24).^24^

Patients were approached during dialysis sessions and received oral and written information about the study from nephrologists or nurse practitioners. Participation was based on nonopposition. Questionnaires were completed electronically during the dialysis session. This approach aimed to assess patients’ perception of their participation in health care decisions and to identify factors associated with this participation.

### Participants

Eligible participants were adults (18 years or older) receiving maintenance hemodialysis for at least 3 months who were able to understand French and provide informed consent. Patients were excluded if they had insufficient French language proficiency, cognitive or psychiatric disorders impairing participation, inability to provide informed consent, or were under legal protection (including guardianship or curatorship).

### Sociodemographic and Clinical Variables

Collected variables included age, sex, marital status, employment status, education level, place of residence, dialysis setting, and dialysis unit type. Educational attainment was categorized as without a diploma, high school level, Baccalaureate (high school completion), or university level. Social deprivation was assessed using the EDI assigned at the dialysis unit level. EDI values corresponded to the geographic territories served by the dialysis units and represented the mean deprivation score of the municipalities whose residents receive dialysis in that unit. Because dialysis care in the study region is organized on a territorial basis, patients are typically treated in dialysis units serving their area of residence. Higher EDI values indicate greater socioeconomic deprivation, and the variable was analyzed as a continuous contextual indicator. Additional contextual variables included type of dialysis autonomy, type of medical decision (dialysis-related, medication-related, or related to comorbid conditions), and the category of health care professional involved in the decision-making process (nephrologist, nurse practitioner, dialysis nurse, or multidisciplinary team).

### Outcome Measures

The primary outcome was patients' perceived level of SDM, measured using the French version of the nine-item SDM Questionnaire (SDM-Q-9) originally developed and validated by Kriston *et al*.^25^ (see Supplemental Table 1). This self-administered questionnaire assessed patient's perceptions of their involvement in clinical decision making over the preceding 15 days. Participants were instructed to identify a specific medical decision that had occurred within the preceding 15 days, as specified in the SDM-Q-9 instructions, and to answer all items with reference to that decision only. The study inclusion period corresponded to the recruitment window and did not restrict the recall period assessed by the questionnaire. The selected decision was first recorded in the questionnaire (“Please indicate which health complaint/problem/illness the consultation was about” and “Please indicate which decision was made”), and all SDM-Q-9 items were answered with reference to this index decision. For analytical purposes, reported decisions were subsequently categorized into three groups: dialysis session–related decisions, medication-related decisions, and decisions related to the management of comorbid conditions. Each item is scored on a five-point Likert scale. Raw SDM-Q-9 scores (range 0–45) were converted to a standardized 0–100 scale, with higher scores indicating greater perceived involvement, and all analyses were performed using the standardized 0–100 score. Item-level descriptive analyses of the SDM-Q-9 responses were also performed. Patients additionally reported the health concern discussed, type of decision made, and the health care professionals involved in the decision-making process.

### HL

HL was assessed using the validated French version of the functional, communicative, and critical health literacy (FCCHL) scale (Supplemental Table 2).^26^ The FCCHL comprises 14 items across three dimensions: functional (five items), communicative (five items), and critical literacy (four items). Each item is rated on a five-point Likert scale. For functional HL items, response options were reverse-scored so that higher scores consistently indicated higher HL across all dimensions. For each dimension, scores were calculated by averaging the item scores within that dimension. An overall HL score was calculated as the mean of the three subscale scores. Consistent with prior studies, a mean domain score ≤4 was interpreted as indicating limited HL for descriptive purposes.^27,28^ For regression analyses, communicative and critical HL domain scores were included as continuous variables to capture dimensions most relevant to patient–clinician communication and decision making.

### Statistical Analysis

Descriptive statistics are presented as mean±SD or median (interquartile range; [Q1–Q3]) for continuous variables and as frequencies and percentages (%) for categorical variables. Internal consistency of the SDM-Q-9 and FCCHL scales was assessed in the study sample using Cronbach *α*. Primary exposures were defined *a priori* as HL (FCCHL scores), socioeconomic deprivation (EDI), and dialysis unit type. Dialysis autonomy and employment status were included as adjustment covariates in the multivariable regression models. Subgroup analyses explored differences in SDM-Q-9 scores according to demographic, clinical, and contextual characteristics. Univariable analyses included Pearson correlation coefficients for continuous variables and ANOVA or Kruskal–Wallis tests for categorical variables, as appropriate. Candidate variables considered for multivariable analysis included demographic characteristics (age, sex, employment status), the global score of HL and HL domains (functional, communicative, and critical), socioeconomic deprivation (EDI), dialysis autonomy, and dialysis unit type. Variables retained in the final model were selected on the basis of the theoretical relevance and potential collinearity considerations.

Multivariable linear regression analyses were conducted to identify independent predictors of SDM-Q-9 scores. Logistic regression models using dichotomized SDM-Q-9 scores were also explored. Multicollinearity was assessed before model interpretation. Dialysis unit type and dialysis autonomy were conceptually related variables; therefore, potential multicollinearity between these predictors was specifically evaluated during model diagnostics. Model assumptions were evaluated using residual diagnostics, including inspection of residual distributions, the Durbin–Watson statistic, and influence analysis using Cook's distance. A two-sided *P* value <0.05 was considered statistically significant. Missing data were minimal, and analyses were performed using complete-case data with *R* software.

### Ethical Considerations

All participants received oral and written information and provided informed consent before participation. The study was approved by the *Comité de Protection des Personnes Île-de-France* (ID RCB: 2024-A00946-41) and declared to the French Data Protection Authority (*Commission Nationale de l’Informatique et des Libertés*). The study was conducted in accordance with French Public Health Code Article L.1121-1 and the category 3 research involving human participants (non-interventional research with minimal or no risk) research methodology.

## Results

### Study Population

**Figure 1:**
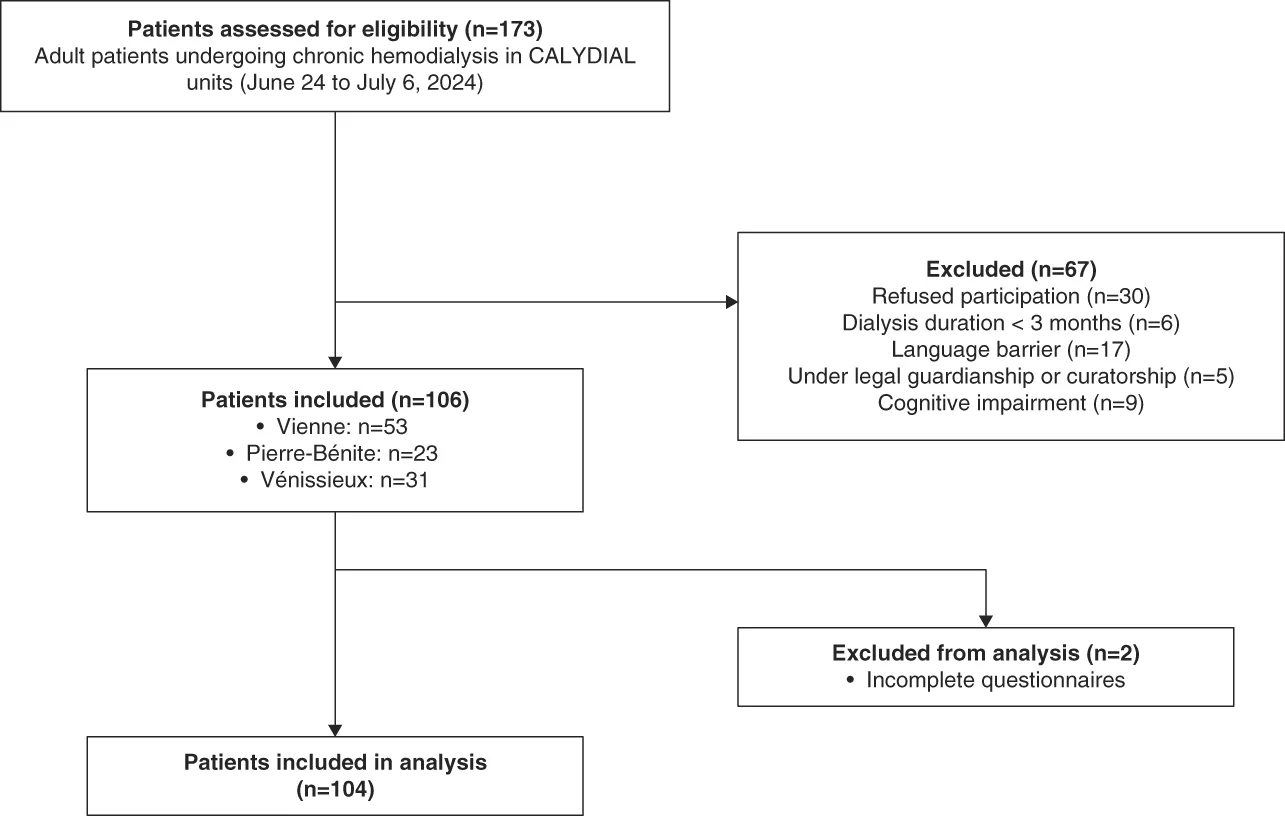
**Study flow chart.** CALYDIAL, Centre Associatif Lyonnais De Dialyse.

Among 173 patients approached, 30 declined participation and 37 did not meet the inclusion criteria. Of the 106 eligible patients, two returned incomplete questionnaires (Figure 1). The response rate among eligible participants was 97%. Participants were mostly male (75%). Forty-four percent were older than 70 years, 48% were aged 50–70 years, and 8% were 30–50 years (Table 1). Most participants were married (58%), followed by being single (18%). Educational attainment was limited, with 24% reporting no diploma and 51% having had a high school-level education. Most participants were retired (64%), 10% were employed, and 26% were unemployed or on medical leave (Table 1).

**Table 1:** Baseline characteristics

Characteristics	Patients, *n* (%)
Sex (male)	78 (75)
**Age group**	
30–50 yr	9 (8)
50–70 yr	49 (48)
Older than 70 yr	46 (44)
**Marital status**	
Married	61 (58)
Living with partner	5 (5)
Single	19 (18)
Widowed	9 (9)
Divorced	6 (6)
Separated	4 (4)
**Education level**	
Without a diploma	25 (24)
High school level	53 (51)
Baccalaureate (high school completion)	10 (10)
University level	16 (15)
**Occupational status**	
Retired	67 (64)
Employed	10 (10)
Employed and on medical leave	14 (14)
Unemployed	13 (12)

### HL and Perceived SDM

**Figure 2:**
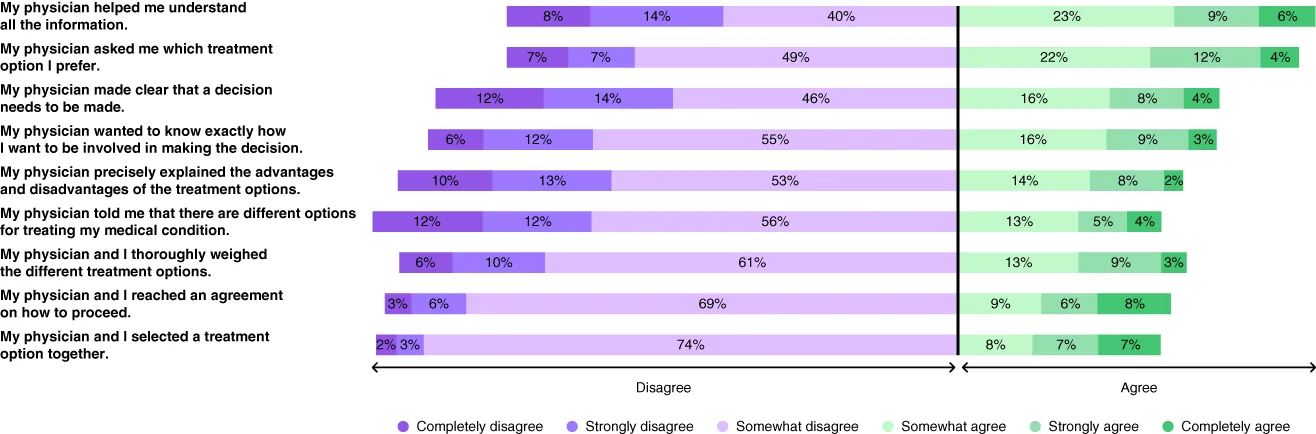
**Distribution of SDM-Q-9 item responses.** SDM-Q-9, nine-item Shared Decision-Making Questionnaire.

Internal consistency was excellent for the SDM-Q-9 (Cronbach *α*=0.94) and good for the FCCHL scale (Cronbach *α*=0.85) in the study sample. On the basis of the FCCHL, the mean total HL score was 54.0±10.7 (possible range, 14 to 70), corresponding to a mean item score of 3.84±0.74 on a 1–5 scale. Overall, 58% of participants had limited HL (mean item score ≤4). Similar proportions of limited HL were observed across functional, communicative, and critical domains (Table 2). The mean SDM-Q-9 score (0–100 scale) was 24.3±26.1 (median of 13.3). Scores were skewed toward lower values. At an item level, the highest proportions of agreement were observed for items related to information provision (38%) and preference elicitation (38%). Agreement was lowest for items related to presentation of treatment options (22%) and joint decision making (22%; Figure 2). Among the decisions reported by participants, most concerned dialysis session–related issues (57%), including dry weight, ultrafiltration, and session duration or frequency. Medication-related decisions accounted for 26% of cases, and decisions related to the management of comorbid conditions accounted for 17%.

**Table 2:** Health literacy scores assessed by the functional, communicative, and critical health literacy scale

Domain	Total Score, mean±SD (Range)	Mean Item Score±SD (1–5 Scale)	Participants with Limited HL (≤4) (%)
**HL**			
Functional	19±5.4 (6–30)	3.83±1.09	51
Communicative	19±4.3 (5–25)	3.88±0.86	51
Critical	15±4.1 (3–25)	3.79±1.04	52
Overall HL	54±10.7 (14–70)	3.84±0.74	58

Total scores represent the sum of item responses within each domain. Mean item scores were calculated by dividing total scores by the number of items in each domain. Limited health literacy was defined as a mean item score ≤4.

HL, health literacy.

### Factors Associated with Perceived SDM

**Figure 3:**
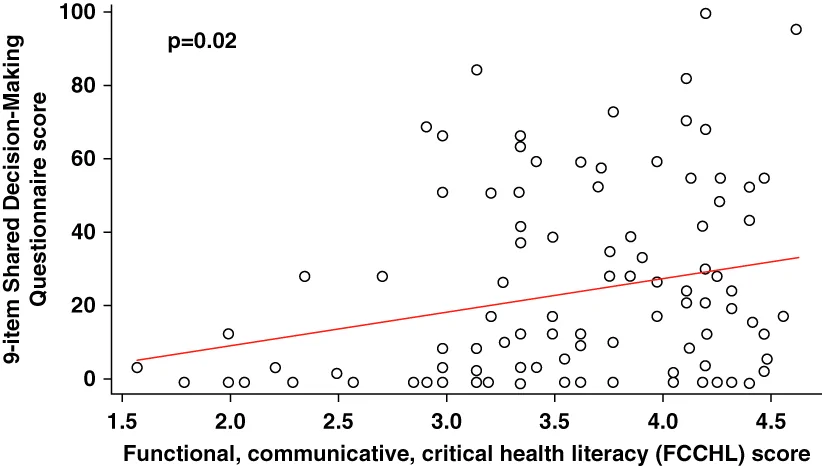
**Correlation between HL and SDM-Q-9 scores.** HL, health literacy.

Higher socioeconomic deprivation was associated with lower SDM-Q-9 scores, and higher communicative and critical HL scores were associated with higher SDM-Q-9 scores. Significant differences in SDM-Q-9 scores were also observed across age group, employment status, dialysis autonomy, dialysis unit type, and decision type (Supplemental Table 3). Specifically, for individual factors, lower SDM-Q-9 scores were observed among participants older than 70 years (*P* = 0.04), retired individuals (*P* = 0.01), widowed participants (*P* = 0.04), and those with limited HL (*P* < 0.05; Table 3). HL scores were positively correlated with SDM-Q-9 scores (*P* = 0.02; Figure 3). By contrast, socioeconomic deprivation was negatively correlated with SDM-Q-9 scores (*r*=−0.243; *P* < 0.05).

**Table 3:** Individual factors associated with shared decision-making perception scores among patients receiving hemodialysis in all CALYDIAL dialysis units

Individual Factors	Mean±SD	Median [Q1–Q3]	*P* Value
**Sex**			
Male	24.5±26.3	13.3 [0.5–42.2]	>0.05
Female	23.8±26.1	16.6 [0–41.1]	
**Marital status**			
Married	21.4±25.8	11.1 [0–37.7]	0.04
Single	25.1±25.3	22.2 [3.3–35.5]	
Widowed	14.3±17.92	8.8 [0–26.6]	
Divorced	33.7±31.9	34.4 [4.4–57.7]	
Living with partner	55.5±30.6	55.5 [53.3–55.5]	
Separated	35±9.8	35.5 [27.7–42.7]	
**Age range (yr)**			
30–50 yr	29.6±22.1	24.4 [17.7–42.2]	0.04
50–70 yr	28.9±27.1	24.4 [4.4–51.1]	
Older than 70 yr	18.4±25.1	5.5 [0–30]	
**Education level**			
High school level	28.5±28.8	17.7 [2.2–53.3]	>0.05
Without a diploma	16.3±21.1	4.4 [0–28.8]	
University level	22.7±21.2	16.6 [10.5–29.4]	
Baccalaureate (high school completion)	24.4±28.1	14.4 [1.1–43.8]	
**Employment status**			
Retired	19.4±25.9	8.8 [0–30]	0.02
Unemployed	32.9±28.8	33.3 [0–51.1]	
Employed and on leave	33.9±22.9	28.8 [13.8–54.4]	
Employed	32.6±23.2	28.8 [14.4–52.7]	
**Living status**			
At home	24.3±26.4	13.3 [0–42.2]	>0.05
Retirement home	25.1±26.7	22.2 [11.1–37.7]	
Hospitalization	24.4±34.5	24.4 [12.2–36.6]	
Rehabilitation service	17.7±0	17.7 [17.7–17.7]	
**HL**			
≤4	22.8±24.9	13.3 [0–41.1]	<0.05
>4	28.6±29	21.1 [2.2–51.1]	

*P* values represent comparisons of shared decision-making perception scores across categories of each variable using ANOVA or Kruskal–Wallis tests, as appropriate. CALYDIAL, Centre Associatif Lyonnais De Dialyse; HL, health literacy.

**Figure 4:**
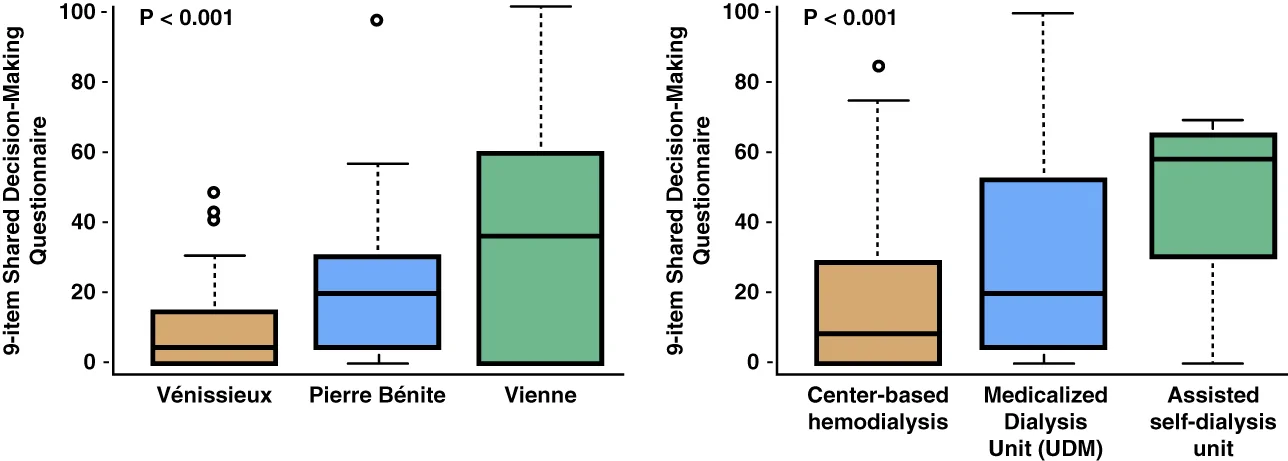
SDM-Q-9 scores according to dialysis unit location and dialysis autonomy.

For contextual factors, perceived SDM differed significantly according to dialysis unit type (*P* = 0.02) and location (*P* < 0.05) (Figure 4 and Table 4). SDM-Q-9 scores were highest in UDM in Vienne and lowest in the Vénissieux dialysis units. SDM-Q-9 scores also varied by decision type (*P* < 0.05), with higher scores observed for decisions related to comorbid conditions and dialysis treatment compared with medication-related decisions. No significant differences in SDM-Q-9 scores were observed according to hemodialysis schedule or health care professional involved in the decision-making process (Table 4).

**Table 4:** External factors associated with shared decision-making perception scores among patients receiving hemodialysis in all CALYDIAL dialysis units

External Factors	*n* (%)	Mean±SD	Median [Q1–Q3]	*P* Value
**Day of hemodialysis, *n*=103**				
Monday, Wednesday, and Friday	61 (59.22)	20.8±22.7	13.3 [0–35.5]	>0.05
Tuesday, Thursday, and Saturday	42 (40.78)	29.3±30	13.3 [3.3–54.4]	
**Dialysis unit type, *n*=104**				
Center-based hemodialysis	48 (46.15)	18.59±23.3	9 [0–33.3]	0.02
UDM	48 (46.15)	27.92±27.9	18 [4.4–51.7]	
Self-dialysis unit	8 (7.7)	44.17±27.2	54 [38.9–61.1]	
**Dialysis unit location, *n*=104**				
Vienne center-based unit	29 (27.9)	20.6±25.3	8.8 [0.0–37.7]	<0.05
Vienne self-dialysis unit	8 (7.7)	44.1±27.1	54.4 [38.8–61.1]	
Vienne UDM	13 (12.5)	54.3±27.6	55.5 [51.1–68.8]	
Vénissieux center-based unit	19 (18.3)	12.5±15.2	6.6 [0.0–20.0]	
Vénissieux UDM	10 (9.6)	9.1±12.5	4.4 [0.0–12.7]	
Pierre Bénite UDM	25 (24.0)	21.7±22.5	17.7 [4.4–28.8]	
**Decision type, *n*=104**				
Dialysis treatment	59 (56.73)	25.8±24.9	22.2 [3.3–42.2]	<0.05
Medication-related	27 (26.0)	12.8±17.9	4.4 [0–16.6]	
Comorbid conditions-related	18 (17.3)	36.6±33.9	33.3 [0–66.6]	
**Health care professional involved, *n*=102**				0.53
Nephrologists	69 (67.6)	25±27.3	13 [0–42.2]	0.53
Nurse practitioners	7 (6.9)	26±23	23 [6.11–51.1]	
Health care team	10 (9.8)	26±22	22 [6.7–51.1]	
Nurses	16 (15.7)	34±27.6	24 [16–55.6]	

Mean±SD and median [interquartile range] are presented. *P* values represent comparisons of shared decision-making perception scores across categories of each variable using ANOVA or Kruskal–Wallis tests, as appropriate. Dialysis unit location reflects both geographic area and organizational structure; analyses are unadjusted. CALYDIAL, Centre Associatif Lyonnais De Dialyse; UDM, medicalized dialysis unit.

### Multivariable Analysis

In multivariable linear regression analyses, higher socioeconomic deprivation remained independently associated with lower SDM-Q-9 scores (*β*=−1.12 per unit increase in EDI; 95% confidence interval [CI; −2.05 to −0.19]; *P* = 0.02; Table 5). Higher communicative (*β*=2.48; 95% CI [0.89 to 4.07]; *P* = 0.003) and critical HL scores (*β*=3.01; 95% CI [1.12 to 4.90]; *P* = 0.002) were independently associated with higher SDM-Q-9 scores. Dialysis unit type and dialysis autonomy also remained independently associated with perceived SDM. Employment status was independently associated with SDM-Q-9 scores, with higher scores observed among employed participants compared with those not employed (*β*=7.96; 95% CI, [2.21 to 13.71]; *P* = 0.007). The final model explained 34.2% of variance in the SDM-Q-9 scores (adjusted *R*^2^=0.342; Table 5).

**Table 5:** Multivariable linear regression analysis of factors associated with perceived shared decision making (nine-item SDM Questionnaire score)

Variable	*β* Coefficient [95% CI]	*P* Value
Socioeconomic deprivation (EDI score)	−1.12 [−2.05 to−0.19]	0.02
Communicative HL (per 1-point increase)	+2.48 [0.89 to 4.07]	<0.01
Critical HL (per 1-point increase)	+3.01 [1.12 to 4.90]	<0.01
Dialysis unit type^a^	+8.75 [3.10 to 14.40]	<0.01
Dialysis autonomy^b^	+6.42 [1.58 to 11.26]	0.01
Employment status (employed versus not employed)	+7.96 [2.21 to 13.71]	0.01

Adjusted *R*^2^=0.342, number of observations=104. CI, confidence interval; EDI, European Deprivation Index; HL, health literacy; SDM, shared decision-making.

a Dialysis unit type entered as a categorical variable.

b Dialysis autonomy was defined according to dialysis setting, with self-dialysis units considered autonomous, medicalized dialysis units considered semiautonomous, and center-based considered fully assisted.

## Discussion

In this multicenter cross-sectional study, we observed generally low levels of perceived SDM in routine hemodialysis care. To our knowledge, this is among the first studies to assess patients' perceptions of SDM over a short, recent time frame encompassing a broad range of everyday decisions in chronic hemodialysis care. Our findings highlight substantial heterogeneity in perceived SDM and underscore the importance of individual, social, and organizational factors associated with patients' involvement in decision making, particularly HL and socioeconomic deprivation, and care structure.

The mean SDM-Q-9 score observed in our cohort (24.3/100) indicates a low level of perceived patient involvement compared with scores reported in nondialysis populations and in studies focusing on major renal treatment decisions, such as dialysis modality choice and advanced care planning.^29,30^ Several of these studies had a high risk of bias and did not specifically include patients receiving hemodialysis. Differences in study populations, decision types, and clinical contexts likely contribute to the variations in findings. Unlike prior studies that examined singular, high-stakes decisions, our study focused on routine clinical decisions occurring during ongoing dialysis care which may be perceived as more clinician-driven, particularly in highly protocolized settings like center-based hemodialysis.

At the item level, patients most frequently reported agreement with items related to information provision and elicitation of preferences, whereas agreement was lowest for the presentation of alternative treatment options and joint decision making. This pattern suggests that while information exchange may occur, the deliberative and collaborative components central to SDM are often limited in routine dialysis encounters. These findings are consistent with previous research indicating that information provision alone does not ensure meaningful SDM.^31^

In our study, older age and lower employment status were associated with lower perceived SDM in univariable analyses. These findings are consistent with prior research showing that older patients tend to adopt more passive roles in clinical decision making, often deferring to health care professionals,^5,32^ influenced by generational attitudes toward medical authority and age-related cognitive or sensory limitations.^33^

HL was associated with perceived SDM. More than half of the participants exhibited limited HL, and higher HL scores were associated with higher SDM-Q-9 scores. Notably, communicative and critical HL were independently associated with perceived SDM, whereas functional HL was not. This suggests that beyond basic comprehension skills, patients' ability to communicate effectively with health care professionals, interpret information, and critically appraise treatment options plays a central role in meaningful participation in decision making.^20^ These results are consistent with conceptual models of SDM that emphasize bidirectional communication and deliberation as essential components of PCC.^11,12,23^

Socioeconomic deprivation, measured at the dialysis unit level using the EDI, was independently associated with lower perceived SDM. This association persisted after adjustment for individual characteristics, suggesting that contextual and structural factors contribute to disparities in patient involvement. Patients treated in more socially deprived areas may experience cumulative barriers, including lower HL, reduced self-efficacy, and communication mismatches with health care professionals. These findings are consistent with broader evidence indicating that socially disadvantaged populations are less likely to engage actively in health care decisions.^12,15,18^ Given the exploratory design and moderate sample size, the model was intended to identify explanatory associations rather than provide predictive estimates.

Care structure was also associated with differences in SDM perception. Dialysis unit type and patient autonomy remained independently associated with SDM-Q-9 scores, indicating that organizational environments may facilitate or hinder patient involvement. Dialysis unit type and dialysis autonomy represent related organizational dimensions, and some degree of structural collinearity between these variables may be present. Self-dialysis and medicalized units may provide conditions more conducive to individualized communication and patient engagement, although further research is needed to better understand the mechanisms underlying these differences. Perceived SDM also varied according to decision type, with higher scores observed for decisions related to dialysis parameters and comorbidity management compared with medication-related decisions. This may reflect greater familiarity with dialysis-related issues, which are discussed regularly during treatment sessions, whereas medication decisions may be perceived as more complex or physician-driven.^8,9^ SDM-Q-9 scores did not differ according to the health care professional involved in the decision-making process, suggesting that, from the patient perspective, SDM is shaped more by the interaction process and care environment than by professional role alone. The adjusted *R*^2^ of 0.342 indicates that a substantial proportion of variability in perceived SDM remains unexplained. This finding is expected given the multidimensional nature of SDM, which may reflect psychological, relational, and experiential factors that were not measured in this study. Variables such as patient activation and self-efficacy, depressive symptoms and psychological distress, previous health care experiences, trust in health care professionals, and clinician communication style may contribute to this residual variability. Future studies integrating psychosocial measures may help better characterize these associations.

Our findings suggest that SDM remains insufficiently implemented in routine hemodialysis care, particularly among older patients, those with limited HL, and those treated in socially deprived settings. Interventions aimed at improving SDM could therefore extend beyond individual physician behavior and address broader organizational and relational barriers to patient engagement, as highlighted in prior reviews of SDM implementation.^13^ Training nephrology teams in HL-sensitive communication strategies and SDM techniques may enhance patient engagement. The development of tailored decision-support tools adapted to the needs of patients receiving hemodialysis, particularly those with limited communicative and critical HL, may also support more meaningful participation in decision making.^34,35^ Integrating nurse practitioners or other trained professionals as facilitators of SDM and promoting structured, multidisciplinary discussions could further ensure that patients' values and preferences are systematically incorporated into care decisions.^36^

Several limitations should be considered. The cross-sectional design precludes causal inference, and the relatively small sample size may limit generalizability; formal sensitivity analyses were not performed. The exclusion of patients with language barriers, cognitive impairment, or legal guardianship may limit applicability to more vulnerable populations and introduce selection bias toward individuals with greater cognitive and communicative capacity, potentially influencing perceived SDM and underestimated social gradients. The cohort, predominantly older with low educational attainment and a higher proportion of men than national populations, may limit representativeness. Psychosocial factors, such as patient activation, self-efficacy, and social support, were not assessed, although they may mediate the relationship between HL and SDM. The SDM-Q-9 was applied to heterogeneous routine decisions, which may have influenced responses. Dialysis vintage beyond the ≥3-month eligibility threshold was not recorded, and residual confounding related to treatment experience cannot be excluded. Clinical complexity and caregiver involvement were not assessed. Finally, the analysis reflects only patients' perceptions of SDM; incorporating physicians' perspectives and longitudinal designs could provide a more comprehensive understanding of decision-making processes.

In this study, perceived SDM during routine maintenance hemodialysis was low and heterogeneous. HL, particularly communicative and critical dimensions, together with socioeconomic deprivation, dialysis autonomy, and care structure, were associated with differences in perceived SDM. Addressing HL-related communication barriers and organizational factors may strengthen SDM and promote PCC in chronic dialysis settings. Further multicenter and longitudinal studies are needed to confirm these findings and evaluate interventions designed to enhance patient engagement and SDM in routine renal care.

## Supplementary Material

## Data Availability

Original data generated for the study will be made available upon reasonable request to the corresponding author. Data Type: Raw Data/Source Data; Survey Data; Statistical Analysis Plan. Reason for Restricted Access: Deidentified individual participant data are not publicly available but may be shared upon reasonable request to the corresponding author, subject to institutional and ethical approval.

## Declarative Statements

This study includes clinical experimentation and received Institutional Review Board or Ethics Committee approval. All patients provided written informed consent. This study includes clinical experimentation and complies with the Declaration of Helsinki.

## Supplemental Material

Supplemental Table 1. SDM-Q-9.

Supplemental Table 2. FCCHL Scale (FCCHL/HLS14).

Supplemental Table 3. Univariate associations with SDM-Q-9 score.
