# Supplementary material for: Perceived Shared Decision Making during Maintenance Hemodialysis: Associations with Health Literacy, Social Deprivation, and Care Setting
Source: Kidney360. 2026 May 4;7(8):1815–24. doi: 10.34067/KID.0000001214 (PMC13567900; doi:10.34067/KID.0000001214)
Supplement: Supplementary file 1 [file kidney360-7-1815-s001.pdf]

ASN American Society of Nephrology JASN Journal of the American Society of Nephrology KIDNEY360 Accessing Our World From Every Angle CJASN Clinical Journal of the American Society of Nephrology

ASN Journal Disclosure Form

As per ASN journal policy, I have disclosed any financial relationships or commitments I have held in the past 36 months as included below. I have listed my Current Employer below to indicate there is a relationship requiring disclosure. If no relationship exists, my Current Employer is not listed.

A. Beaudoin reports the following:
Employer: ISS MSP Grigny sur Rhone; Honoraria: VIFOR; OPTI'MRC; and Advisory or Leadership Role: HAS expert visiteur; CEPPRAAL audit.

I understand that the information above will be published within the journal article, if accepted, and that failure to comply and/or to accurately and completely report the potential financial conflicts of interest could lead to the following: 1) Prior to publication, article rejection, or 2) Post-publication, sanctions ranging from, but not limited to, issuing a correction, reporting the inaccurate information to the authors' institution, banning authors from submitting work to ASN journals for varying lengths of time, and/or retraction of the published work.

Name: Agnes Beaudoin
Manuscript ID: K360-2026-000092R1
Manuscript Title: "Perceived shared decision-making during maintenance hemodialysis: Associations with health literacy, social deprivation, and care setting
Date of Completion: March 12, 2026
Disclosure Updated Date: March 12, 2026

ASN American Society of Nephrology
JASN Journal of the American Society of Nephrology
KIDNEY360 Accessing Our World From Every Angle
CJASN Clinical Journal of the American Society of Nephrology

ASN Journal Disclosure Form

As per ASN journal policy, I have disclosed any financial relationships or commitments I have held in the past 36 months as included below. I have listed my Current Employer below to indicate there is a relationship requiring disclosure. If no relationship exists, my Current Employer is not listed.

N. EL KALAI has nothing to disclose.

I understand that the information above will be published within the journal article, if accepted, and that failure to comply and/or to accurately and completely report the potential financial conflicts of interest could lead to the following: 1) Prior to publication, article rejection, or 2) Post-publication, sanctions ranging from, but not limited to, issuing a correction, reporting the inaccurate information to the authors' institution, banning authors from submitting work to ASN journals for varying lengths of time, and/or retraction of the published work.

Name: Najoi EL KALAI
Manuscript ID: K360-2026-000092R1
Manuscript Title: Perceived shared decision-making during maintenance hemodialysis: Associations with health literacy, social deprivation, and care setting
Date of Completion: March 17, 2026
Disclosure Updated Date: March 17, 2026

ASN American Society of Nephrology | JASN Journal of the American Society of Nephrology | KIDNEY360 Accessing Our World From Every Angle | CJASN Clinical Journal of the American Society of Nephrology

ASN Journal Disclosure Form

As per ASN journal policy, I have disclosed any financial relationships or commitments I have held in the past 36 months as included below. I have listed my Current Employer below to indicate there is a relationship requiring disclosure. If no relationship exists, my Current Employer is not listed.

F. Guebre Egziabher reports the following:
Employer: Hospices Civils de Lyon; Université Lyon-1; Consultancy: ALNYLAM; and Other Interests or Relationships: Member of society francophone de nephrologie dialyse transplantation; Member of ERA/EDTA; member of ISN, Member of EthioAmerican doctors group; Board member of french society of value based healthcare SFVS.

I understand that the information above will be published within the journal article, if accepted, and that failure to comply and/or to accurately and completely report the potential financial conflicts of interest could lead to the following: 1) Prior to publication, article rejection, or 2) Post-publication, sanctions ranging from, but not limited to, issuing a correction, reporting the inaccurate information to the authors' institution, banning authors from submitting work to ASN journals for varying lengths of time, and/or retraction of the published work.

Name: Fitsum Guebre Egziabher
Manuscript ID: K360-2026-000092R1
Manuscript Title: Perceived shared decision-making during maintenance hemodialysis: Associations with health literacy, social deprivation, and care setting.
Date of Completion: March 11, 2026
Disclosure Updated Date: March 11, 2026

ASN
American Society of Nephrology
JASN
Journal of the American Society of Nephrology
KIDNEY360
Accessing Our World From Every Angle
CJASN
Clinical Journal of the American Society of Nephrology

ASN Journal Disclosure Form

As per ASN journal policy, I have disclosed any financial relationships or commitments I have held in the past 36 months as included below. I have listed my Current Employer below to indicate there is a relationship requiring disclosure. If no relationship exists, my Current Employer is not listed.

A. Guerraoui reports the following:
Employer: Calydial; and Speakers Bureau: fresinus medical care; Astra zeneka.

I understand that the information above will be published within the journal article, if accepted, and that failure to comply and/or to accurately and completely report the potential financial conflicts of interest could lead to the following: 1) Prior to publication, article rejection, or 2) Post-publication, sanctions ranging from, but not limited to, issuing a correction, reporting the inaccurate information to the authors' institution, banning authors from submitting work to ASN journals for varying lengths of time, and/or retraction of the published work.

Name: Abdallah Guerraoui
Manuscript ID: K360-2026-000092R1
Manuscript Title: Perceived shared decision-making during maintenance hemodialysis: Associations with health literacy, social deprivation, and care setting
Date of Completion: March 16, 2026
Disclosure Updated Date: March 16, 2026

ASN
American Society of Nephrology
JASN
Journal of the American Society of Nephrology
KIDNEY360
Accessing Our World From Every Angle
CJASN
Clinical Journal of the American Society of Nephrology

ASN Journal Disclosure Form

As per ASN journal policy, I have disclosed any financial relationships or commitments I have held in the past 36 months as included below. I have listed my Current Employer below to indicate there is a relationship requiring disclosure. If no relationship exists, my Current Employer is not listed.

J. Haesebaert reports the following:
Employer: Hospices Civls de Lyon

I understand that the information above will be published within the journal article, if accepted, and that failure to comply and/or to accurately and completely report the potential financial conflicts of interest could lead to the following: 1) Prior to publication, article rejection, or 2) Post-publication, sanctions ranging from, but not limited to, issuing a correction, reporting the inaccurate information to the authors' institution, banning authors from submitting work to ASN journals for varying lengths of time, and/or retraction of the published work.

Name: Julie Haesebaert
Manuscript ID: K360-2026-000092R1
Manuscript Title: Perceived shared decision-making during maintenance hemodialysis: Associations with health literacy, social deprivation, and care setting,
Date of Completion: March 16, 2026
Disclosure Updated Date: March 16, 2026
