# Supplementary material for: Perceived Shared Decision Making during Maintenance Hemodialysis: Associations with Health Literacy, Social Deprivation, and Care Setting
Source: Kidney360. 2026 May 4;7(8):1815–24. doi: 10.34067/KID.0000001214 (PMC13567900; doi:10.34067/KID.0000001214)
Supplement: Supplementary file 2 [file kidney360-7-1815-s002.pdf]

Supplemental Material

Supplemental Table 1. 9-item Shared Decision-Making Questionnaire (SDM-Q-9)

Item	Questionnaire item	Likert response scale*
**Instruction**	Participants were first asked to identify a medical decision that had occurred within the previous 15 days and to indicate the health problem and decision taken. All SDM-Q-9 items were then answered with reference to this selected decision.	-
**1**	My physician made clear that a decision needs to be made.	1 to 6
**2**	My physician wanted to know exactly how I want to be involved in making the decision.	1 to 6
**3**	My physician told me that there are different options for treating my medical condition.	1 to 6
**4**	My physician precisely explained the advantages and disadvantages of the treatment options.	1 to 6
**5**	My physician helped me understand all the information.	1 to 6
**6**	My physician asked me which treatment option I prefer.	1 to 6
**7**	My physician and I thoroughly weighed the different treatment options.	1 to 6
**8**	My physician and I selected a treatment option together.	1 to 6
**9**	My physician and I reached an agreement on how to proceed.	1 to 6

*Response scale: 1=Completely disagree; 2=Strongly disagree; 3=Somewhat disagree; 4=Somewhat agree; 5=Strongly agree and 6=Completely agree. Raw SDM-Q-9 scores (0–45) were linearly transformed to a 0–100 scale for analysis.

Supplemental Table 2. Functional, Communicative and Critical Health Literacy Scale (FCCHL/HLS14)

Dimension	Item	Questionnaire item	Likert response scale*
**Functional**	HL1†	There are words that I do not know.	5 to 1
**Functional**	HL2†	The print is too small for me (even when I wear glasses).	5 to 1
**Functional**	HL3†	The content is too difficult for me.	5 to 1
**Functional**	HL4†	It takes me a long time to read it.	5 to 1
**Functional**	HL5†	I need help to read it.	5 to 1
**Communicative**	HL6	I gather information from several sources.	1 to 5
**Communicative**	HL7	I extract from the available information what is relevant to me.	1 to 5
**Communicative**	HL8	I understand the information obtained.	1 to 5
**Communicative**	HL9	I express my opinion about my illness to my doctor and to people around me (family or friends).	1 to 5
**Communicative**	HL10	I use the information obtained to change my daily life.	1 to 5
**Critical**	HL11	I consider whether the information applies to my situation.	1 to 5
**Critical**	HL12	I consider whether the information is credible.	1 to 5
**Critical**	HL13	I ask someone or verify whether the information is correct.	1 to 5
**Critical**	HL14	I seek information in order to make decisions about my health by myself.	1 to 5

Response scale: 1 = Never; 2 = Rarely; 3 = Sometimes; 4 = Often; 5 = Always. †Functional health literacy items (HL1–HL5) were reverse coded so that higher scores indicate higher health literacy.

Supplemental Table 3. Univariate associations with SDM-Q-9 score

Variable	Type of variable	Statistical test	**Test statistic**	p-value
**Socioeconomic deprivation (EDI score)**	Continuous	Pearson	r=–0.243	0.014
**Health literacy (communicative)**	Continuous	Pearson	r=0.268	<0.001
**Health literacy (critical)**	Continuous	Pearson	r=0.280	<0.001
FCCHL total score	Continuous	Pearson	r=0.225	0.003
Age range (years)	Categorical	ANOVA	F=3.21	0.045
Marital status	Categorical	ANOVA	F=2.50	0.035
Employment status	Categorical	ANOVA	F=4.10	0.019
Dialysis autonomy	Categorical	ANOVA	F=4.03	0.021
Dialysis unit type	Categorical	ANOVA	F=7.43	<0.001
Decision type	Categorical	ANOVA	F=3.31	0.041

SDM-Q-9; 9-item Shared Decision-Making Questionnaire, FCCHL; Functional, Communicative and Critical Health Literacy scale. All analyses are unadjusted. Pearson correlation coefficients were used for continuous variables and analysis of variance (ANOVA) for categorical variables. All analyses were performed using complete-case data (n=104). A two-sided p value <0.05 was considered statistically significant.
